# The construction of a high-density linkage map for identifying SNP markers that are tightly linked to a nuclear-recessive major gene for male sterility in *Cryptomeria japonica *D. Don

**DOI:** 10.1186/1471-2164-13-95

**Published:** 2012-03-16

**Authors:** Yoshinari Moriguchi, Tokuko Ujino-Ihara, Kentaro Uchiyama, Norihiro Futamura, Maki Saito, Saneyoshi Ueno, Asako Matsumoto, Naoki Tani, Hideaki Taira, Kenji Shinohara, Yoshihiko Tsumura

**Affiliations:** 1 Department of Forest Genetics, Forestry and Forest Products Research Institute, Tsukuba, Ibaraki 305-8687, Japan; 2 Department of Molecular and Cell Biology, Forestry and Forest Products Research Institute, Tsukuba, Ibaraki 305-8687, Japan; 3 Toyama Prefectural Agricultural Forestry and Fishieries Research Center, Forestry Research Institute, Yoshimine 3, Tateyama-cho, Nakashinkawagun, Toyama 930-1362, Japan; 4 Forestry Division, Japan International Research Center for Agricultural Sciences, Ohwashi, Tsukuba, Ibaraki 305-8686, Japan; 5 Graduate School of Science and Technology, Niigata University, Igarashi 2-nocho, Niigata 950-2101, Japan

## Abstract

**Background:**

High-density linkage maps facilitate the mapping of target genes and the construction of partial linkage maps around target loci to develop markers for marker-assisted selection (MAS). MAS is quite challenging in conifers because of their large, complex, and poorly-characterized genomes. Our goal was to construct a high-density linkage map to facilitate the identification of markers that are tightly linked to a major recessive male-sterile gene (*ms1*) for MAS in *C. japonica*, a species that is important in Japanese afforestation but which causes serious social pollinosis problems.

**Results:**

We constructed a high-density saturated genetic linkage map for *C. japonica *using expressed sequence-derived co-dominant single nucleotide polymorphism (SNP) markers, most of which were genotyped using the GoldenGate genotyping assay. A total of 1261 markers were assigned to 11 linkage groups with an observed map length of 1405.2 cM and a mean distance between two adjacent markers of 1.1 cM; the number of linkage groups matched the basic chromosome number in *C. japonica*. Using this map, we located *ms1 *on the 9^th ^linkage group and constructed a partial linkage map around the *ms1 *locus. This enabled us to identify a marker (hrmSNP970_sf) that is closely linked to the *ms1 *gene, being separated from it by only 0.5 cM.

**Conclusions:**

Using the high-density map, we located the *ms1 *gene on the 9^th ^linkage group and constructed a partial linkage map around the *ms1 *locus. The map distance between the *ms1 *gene and the tightly linked marker was only 0.5 cM. The identification of markers that are tightly linked to the *ms1 *gene will facilitate the early selection of male-sterile trees, which should expedite *C. japonica *breeding programs aimed at alleviating pollinosis problems without harming productivity.

## Background

High-density linkage maps facilitate not only the understanding of genome structure and comparative genomic studies, but also quantitative trait loci (QTL) mapping and the construction of partial linkage maps around target loci to develop markers for marker-assisted selection (MAS). MAS is an effective method for accelerating the screening of target genes using tightly-linked molecular markers. Effective MAS for QTLs or specific genes has been reported in crops such as barley [1], rice [2] and tomato [3]. However, it is difficult to employ MAS in conifers, primarily because their genomes are very big and complex and are largely uncharacterized. Indeed, very few DNA markers linked to major genes have been reported in conifers; the only ones of note are some dominant markers linked to a major dominant resistance gene in *Pinus lambertiana *[4,5], *P. monticola *[6], *P. taeda *[7], and *P. thunbergii *[8,9].

Sugi (*Cryptomeria japonica *D. Don) is an allogamous, diploid, wind-pollinated conifer species with a haploid chromosome number (n) of 11 (2n = 22). Using flow cytometry, the DNA content of its haploid cells was estimated to be 11.045 pg/C [10], which corresponds to a haploid genome size of 10.8 Gb as calculated using the following expression: genome size (bp) = 0.978 × 10^9 ^× DNA content (pg) [11]. *C. japonica *is frequently used for commercial afforestation in Japan, and about 45% of all the man-made forests of Japan are composed of this species. However, since the 1970s, the incidence of *C. japonica *pollinosis in Japan has risen in line with the increasing number of man-made *C. japonica *forests [12]. Today, *C. japonica *pollinosis is a serious social problem in Japan, affecting almost 20% of the population. In 1992, a genetically male-sterile *C. japonica *tree whose sterility is determined by a major recessive gene (*ms1*) was found in Toyama prefecture [13,14]; this gene is expected play an important role in breeding for reduced pollen dispersal. Since the discovery of this male-sterile individual, considerable effort has been expended on characterizing male sterility in *C. japonica*, identifying male-sterile and plus-trees, creating artificial crosses between male-sterile and plus-trees, and propagating male-sterile trees [15]. Male-sterile trees are currently identified by direct inspection of the male strobili using a magnifying glass or a microscope. Conversely, plus-trees that are heterozygous for *ms1 *have been identified by examining segregation data for the progeny arising from artificial crosses; such trees provide important breeding material for seed production while avoiding the problems associated with inbreeding depression. However, these methods are very time-, labour-, and space-intensive; as such, it would be extremely useful to develop a MAS-based method for selecting trees carrying the male-fertile gene.

A composite linkage map for *C. japonica *was constructed using data for two pedigrees, YI and KO [16]. A total of 438 markers were assigned to 11 large linkage groups and some small or non-integrated linkage groups; the total observed map length was 1372.2 cM, and the average marker interval was 3.0 cM. In general, the most efficient way to study the linkage of a given target gene in a mapping population for which no linkage map is available is to start with a high-density linkage map.

In a recent study on spruces, it was found that the Golden Gate single nucleotide polymorphism (SNP) assay system developed by Illumina greatly facilitates the genetic mapping of species whose genomes have not been extensively studied [17]. To design an SNP genotyping array, it is necessary to identify a large number of SNPs. In the case of *C. japonica*, a database of 55,543 expressed sequence tags (ESTs) has been constructed from cDNA libraries obtained from seedlings, inner bark, female strobili, male strobili, pollen, leaves, vegetative buds and heartwood; this database is freely-available on the internet (ForestGEN; http://forestgen.ffpri.affrc.go.jp/en/info_cj.html) [18-23]. A number of cDNA-based sequence-tagged site (STS) markers have been also identified using these ESTs [24-26]. A library of *C. japonica *SNPs suitable for use in a Golden Gate SNP array has been identified [27], and complements the existing *C. japonica *EST databases, which will also be useful in identifying candidate genes associated with male gametophyte development and male sterility on the basis of sequence similarity and microarray expression analysis.

This paper reports a study in which a high-density linkage map for *C. japonica *was constructed and the mapping population was subjected to the GoldenGate genotyping assay. Using information from the high-density linkage map, we mapped the *ms1 *gene and constructed a partial linkage map around the *ms1 *locus. This allowed us to identify SNP markers that are tightly linked to the *ms1 *gene for use in MAS. The paper also includes a discussion of the importance of high-density maps and MAS markers in tree breeding.

## Methods

### Plant materials and DNA extraction

**Figure 1:**
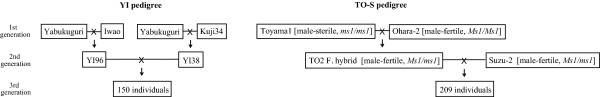
**Two three-generation pedigrees used for linkage mapping in this study**.

A high density linkage map for the YI pedigree of *C. japonica *was constructed using 150 half-sib progenies from crosses between two F_1 _plants (YI96 ('Yabukuguri × Iwao') × YI38 ('Yabukuguri × Kuji34')) (Figure 1) [16]. In addition, to avoid the effects of inbreeding depression, a partial linkage map around the *ms1 *locus (*ms1 *is a recessive male-sterile gene) was constructed for 209 progenies of the TO-S pedigree, which was derived from a cross between a TO2 F_1 _plant and the elite tree Suzu-2 (Figure 1). The TO2 F_1_ plant was a cross between 'Toyama1 [male-sterile, *ms1/ms1*]' and 'Ohara-2 [male-fertile, *Ms1/Ms1*]' and is thus a male-fertile tree that is heterozygous for the *ms1 *gene (*Ms1/ms1*). Suzu-2 is also male-fertile and heterozygous for the *ms1 *gene [male-fertile, *Ms1/ms1*] [28]. Of the 209 TO-S progeny, 142 were male-fertile and 67 were male-sterile. The actual segregation ratio thus deviated significantly from the expected ratio of 3:1 (*X*^2 ^= 5.55, *df *= 1, *P <*0.05); the reason for this is as yet unknown.

Needle tissue was collected from the parents and progeny of both the YI and TO-S pedigrees. Genomic DNA was extracted from individual needles using a modification of the CTAB method [29]. For use in the Illumina GoldenGate assay, the extracted DNA was purified using a genomic DNA purification kit (Promega) and its concentration was standardized (100-200 ng/uL).

### Genetic markers

For the YI pedigree, six kinds of genetic markers were used to construct the linkage map: cleaved amplified polymorphic sequences (CAPS) markers, restriction fragment length polymorphism (RFLP) markers, microsatellite (simple sequence repeat; SSR) markers, EST-derived microsatellite (EST-SSR) markers, amplicon length polymorphism (ALP) markers and single nucleotide polymorphisms (SNP) merkers. The segregation data for 121 CAPSs, 117 RFLPs, 34 SSRs, 1 ALP and 5 SNP markers were obtained in the previous study [16]. In this study, we added 16 EST-SSR markers and 968 SNP markers including 761 gSNPs, 159 hrmSNPs, 33 ssSNPs and 15 meaSNPs to the linkage map for the YI pedigree (refer to latter sections for these names of SNPs). Detailed information on these markers can be found at Sugi Genome Database website (http://www.ffpri.affrc.go.jp/labs/cjgenome/).

### SNP genotyping for randomly-developed markers

A large set of SNP markers was genotyped using the GoldenGate assay; collectively, this set is referred to as "gSNPs". For the YI pedigree, multiplexed genotyping of the gSNP markers was carried out using the 1536-plex GoldenGate array, in accordance with the manufacturer's protocol. A detailed description of the procedures employed and results obtained in the course of discovering these SNPs can be found elsewhere [27]. A total of 0.5-1.0 μg of genomic DNA per sample (at a concentration of 100-200 ng/μl) was used in the GoldenGate assay. To screen the gSNP markers linked to the *ms1 *gene, 17 male-sterile progenies and the parents of TO-S pedigree were also genotyped using this assay, since the gSNP markers found to be monomorphic in the YI pedigree were excluded from linkage mapping in the TO-S pedigree. The GoldenGate assay employs highly multiplexed allele-specific extension methods and universal PCR amplification reactions. The PCR products, which were fluorescently labeled by the incorporation of 5'-labeled primers P1 (Cy3) and P2 (Cy5), were hybridized to capture probes on the beads in the array. The ratio of the fluorescent signals from 2 allele-specific ligation products was used to determine the sample's genotype. Signal intensity data processing, clustering and genotype calling were performed using the genotyping module in the BeadStudio software (Illumina). Genotyping was conducted exclusively on the basis of SNPs with an Illumina GenTrain score in excess of 0.25; the GenTrain score provides a measure of the reliability of SNP detection based on the distribution of genotypic classes. For each analyzed SNP, individual genotypes with an Illumina GenCall score below 0.25 were excluded; the GenCall score provides a measure of the reliability of an individual SNP call relative to the distribution of genotypic classes.

High Resolution Melting (HRM) analysis was also used to obtain SNP genotyping data for linkage map construction; the SNPs identified in this way are henceforth referred to as "hrmSNPs". The development procedure and analyzed condition was reported elsewhere [30]. The linkage map also incorporated EST-SSR markers; details on the use of this data have been reported previously [31].

### Screening and genotyping of candidate genes

Sequence information for genes related to male gametophyte development and sterility were collected by reviewing the literature on other plant species such as *Arabidopsis *[32-42], *Brassica *[43], *Oryza *[44-46], *Nicotiana *[47] and *Petunia *[48]. The sequences from these publications were then compared to the available *C. japonica *EST sequences using TBLASTN (Additional file 1) [49]. In addition, genes related to male-sterility were screened by comprehensive expression analysis using a microarray containing around 366,000 probes derived from 22,882 tentative consensus sequences obtained from ESTs. Genes that exhibited at least a four-fold difference in expression between male-sterile and male-fertile strobili during the end of September and mid-October were selected for further analysis (Futamura et. al, manuscript in preparation). These time points were chosen because they represent the periods immediately before and immediately after male gametogenesis was observed in male-sterile strobili.

To identify SNP markers, PCR primers for the selected candidate genes were designed using the Primer3 software. PCR amplifications were carried out using a Model 9700 GeneAmp PCR system (Applied Biosystems) in reaction mixtures with a total volume of 15 μL containing 20 mmol/L Tris-HCl (pH 8.0), 50 mmol/L KCl, 2 mmol/L MgCl_2_, 0.2 mmol/L of each deoxynucleoside triphosphate, 0.2 μmol/L of each primer, 5 ng template DNA, and 1.0 units of *Taq *polymerase (Promega). The following thermal profile was used: 3 min denaturation at 94°C, followed by 30 cycles of 45 s denaturation at 94°C, 30 s annealing at 55-64°C, and 30 s extension at 72°C, with a final extension step of 72°C for 10 min. Amplification products were separated by electrophoresis in 2% (w/v) agarose gels run in 1 × TAE buffer. The gels were then stained with ethidium bromide and visualized under UV light. Each PCR fragment was sequenced using the BigDye Terminator kit (Applied Biosystems) and ABI Prism 3100 DNA sequencer (Applied Biosystems) to identify SNPs in the parents of the YI pedigree (YI96 and YI38). Primers for both the forward and the backward direction were used. SNP markers associated with genes having significant similarities to genes from other species that are known to be important in male gametophyte development and/or male-sterility were collectively referred to as ssSNPs, while SNP markers associated with genes exhibiting differential expression between male-fertile and male-infertile individuals were collectively referred to as meaSNPs. The ssSNP and meaSNP markers were genotyped by sequencing. For the mapped meaSNP markers, proteins with high similarities to their original ESTs were identified from the NCBI RefSeq database using BLASTX (Additional file 2) [49]

### Construction of the linkage map for the YI pedigree

Linkage analyses were conducted for all hrmSNP (Additional file 3) [30], EST-SSR [31], gSNP [27], ssSNP (Additional file 1) and meaSNP (Additional file 2) markers that exhibited polymorphism in the parents of the YI pedigree. The segregation data for CAPS, RFLP, SSR, SNP, and ALP markers in the previous study [16] were used together with the genotype data obtained in this work to construct a linkage map.

Chi-squared tests were performed for each locus to assess its deviation from the expected Mendelian segregation ratio. Loci exhibiting extreme segregation distortion (*P <*0.001) were excluded from further linkage analysis. All linkage analyses were performed using the JoinMap v3.0 software with the parameter CP (cross-pollination) [50]. During the construction of the maps, markers were assigned to tentative linkage groups using logarithm of odds ratio (LOD) thresholds of 3.0 to 9.0, with increments of 1.0; an LOD threshold of 8.0 was ultimately used when defining groups of markers. Map distances were calculated using the Kosambi mapping function [51]. For the other parameters such as recombination frequency threshold and a ripple value, default settings were used. Images of the linkage groups were drawn using the Mapchart v2.0 software [52].

### Estimation of genome length and map coverage

The observed genome length (*G_o_*) for the linkage map was calculated as the sum of the sizes of the linkage groups. The expected genome length, *G_e_*, was estimated using method 4 of Chakravarti et al. (1991) [53], in which the total length of the linkage groups is multiplied by the factor (m + 1)/(m-1), where m is the number of markers in the linkage groups. The observed map coverage, *C_o_*, is the ratio of the observed and the estimated genome lengths, i.e. *G_o_*/*G_e_*. The expected genome coverage, *C_e_*, was calculated using the equation of Bishop et al. (1983) [54]:

$$C_{e}=1-[\frac{2R}{N+1}\{{\left(1-\frac{X}{2G_{e}}\right)}^{N+1}-(1-\frac{X}{G_{e}})N+1\}+(1-\frac{RX}{G_{e}}\times (1-\frac{X}{G_{e}})N]$$

Here, R is the haploid number of chromosomes, N is the number of positioned loci, X is the maximum observed map distance between two adjacent assigned markers in cM at or above a minimum LOD threshold value of 8.0, and *G_e _*is the estimated genome length.

### Analysis of marker distribution

If the markers were randomly distributed and the genome is divided in N intervals, the number of markers per interval would follow a Poisson distribution with a mean of μ. To determine whether the markers were randomly distributed, all linkage groups were divided into 1, 2, 3, 4, 5, 10 and 15 cM intervals. The number of intervals that contained markers were counted and the average number of markers per interval (μ) was calculated. If the average number of random occurrences per interval is μ, then the probability that *x *markers will fall within a given interval is

$$P\left(x\right)=\frac{e^{-u}{\left(\mu \right)}^{x}}{x!}$$

We compared the actual distribution of markers to that expected for a Poisson distribution using the chi-squared test as described by Kang et al. (2010) [55].

### Localization of *ms1 *gene

Microsatellite markers were used to identify the linkage group on which the target gene is located because of their high polymorphism and straightforward analysis. Thus, to identify the linkage group containing the *ms1 *gene, a total of 19 microsatellite markers on the linkage map (Additional file 4) that were polymorphic in the parents of the TO-S pedigree were genotyped for 48 of the male-sterile progeny of the TO-S pedigree. If markers were linked to *ms1*, they should be significantly deviated from segregation rates expected from parental genotypes. PCR amplifications were carried out using the Model 9700 GeneAmp PCR System (Applied Biosystems). A reaction mixture with a total volume of 8 μL was used, consisting of 1 × Multiplex PCR master mix (Qiagen), fluorescently-labeled forward primers (0.2 μM), reverse primers (0.2 μM), and 5 ng of genomic DNA. The following thermal profile was used: 15 min at 94°C, then 32 cycles of 30 sec at 94°C, 90 sec at 55-62°C, 60 sec at 72°C, followed by 30 min at 72°C; the results were analyzed using a 3100 genetic analyzer (Applied Biosystems). The independence of the segregation of the SSR markers and the *ms1 *gene was investigated using chi-square tests to identify markers linked to the *ms1 *gene.

### The construction of a partial linkage map around the *ms1 *locus

Linkage analysis using EST-SSR markers indicated that the *ms1 *gene was located in the 9^th ^linkage group, which is hereafter referred to as "LG9." The markers in LG9 that exhibited polymorphism in the parents of the TO-S pedigree were therefore used to construct a partial linkage map around the *ms1 *locus. In addition, gSNP markers that exhibited polymorphism in the parents of the TO-S pedigree and whose segregation with the *ms1 *gene deviated significantly from that expected in the absence of linkage (as judged by the chi-squared test, *P <*0.05) in 17 male-sterile progenies were also used in the construction of the partial linkage map.

The markers of LG-9 in the TO-S pedigree except two microsatellite markers and four CAPS markers were genotyped on a BioMark 48.48 Dynamic Array (Fluidigm) using KASPar assays. Primer pairs suitable for the KASPar assays were designed on the basis of the sequences of the relevant markers (gSNP, meaSNP, ssSNP, hrmSNP and CAPS); see Additional file 5. A total of 6.5 ng of genomic DNA per sample (at a concentration of 5 ng/μl) was used for specific target amplification (STA); the KASPar reactions were performed using the STA products after dilution by a factor of 100. The primers were designed and the assays were performed as specified by the manufacturer. The data obtained were analyzed using the Fluidigm SNP Genotyping Analysis software to obtain genotype calls.

Linkage analyses for the TO-S pedigree were performed using the same conditions as were used for the YI pedigree. Loci whose segregation patterns deviated significantly from Mendelian ratios were not excluded from the further linkage analysis in the TO-S pedigree because distortion of loci linked to the *ms1 *gene was expected in this case.

## Results

### Identifying SNP markers associated with candidate genes related to male gametophyte development and male sterility

On the basis of the sequence similarity results, 238 primer pairs were designed for various candidate genes; of these, 141 generated PCR products that could be separated and identified after electrophoresis on a 2% agarose gel. Of these obtained STSs, 36 were polymorphic in the parents of the YI pedigree (Additional file 1).

In the microarray expression analysis, significant differences in expression were observed between trees with male-fertile and male-sterile strobili using probes derived from 32 different tentative consensus sequences. Primer pairs were designed on the basis of these sequences and the 17 meaSNP markers that exhibited polymorphism in the parents of the YI pedigree (Additional file 2).

### Genotyping markers

A total of 1304 gSNP markers from the 1536 SNP array (84.9%) were successfully genotyped, of which 795 exhibited clear segregation within the YI pedigree. In addition to these gSNP markers, 144 CAPSs, 135 RFLPs, 41 microsatellites, 16 EST-SSRs, 173 hrmSNPs (Additional file 3), 6 SNPs, 1 ALP, 36 ssSNPs and 17 meaSNPs markers were also used in mapping. 74 of these markers exhibited significant deviations (as judged by chi-squared tests, *P ≤ *0.001) from the expected Mendelian ratios and were therefore excluded from further linkage analysis; among the excluded markers were 22 gSNPs, 8 hrmSNPs, 20 CAPSs, 13 RFLPs, 6 SSRs, 3 ssSNPs and 2 meaSNPs (Table 1).

**Table 1:** Parameters of the linkage map for the YI pedigree in *C. japonica*

Mapping parameters	YI pedigree
Total number of available markers	1364

Number of distorted markers (*P* < 0.01)	74

Total number of markers without segregation distortion	1290

Number of unlinked markers	11

Total number of assigned markers	1279

Number of positioned markers	1262

Number of gSNP markers	761

Number of hrmSNP markers	159

Number of CAPS markers	121

Number of RFLP markers	117

Number of SSR markers	34

Number of ssSNP markers	33

Number of meaSNP markers	15

Number of EST-SSR markers	16

Number of SNP markers	5

Number of ALP markers	1

Average map density, cM	1.1

Total observed map length *G _o_*, cM	1405.2

Expected map length *G _e_*, cM	1430.6

Observed map coverage *C _o_* (%)	98.2

Expected map coverage *C _e_* (%)	100.0

### Construction of a linkage map for the YI pedigree

**Figure 2:**
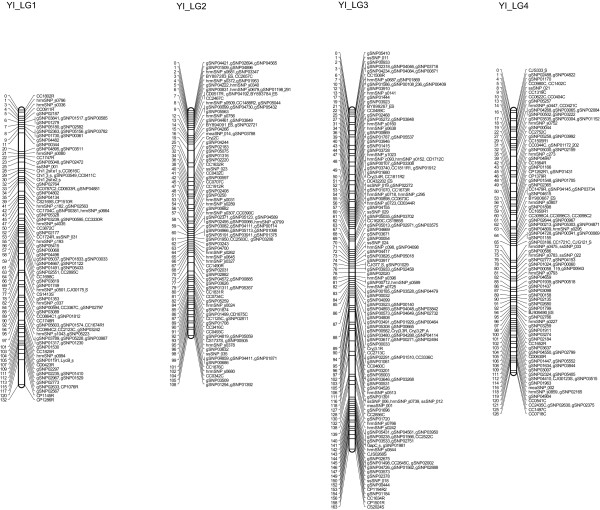
**A linkage map for *C. japonica* derived from the YI pedigree (LG1, LG2, LG3 and LG4)**. Marker names are indicated to the right of the linkage groups. Centimorgan distances (Kosambi) are indicated to the left of each linkage group. Definitions for the labels ssSNP, maeSNP and hrmSNP are provided in the Materials and Methods section. The nature of the other markers is indicated by the last letter of the locus names: C, CAPS; R, RFLP; S, SSR; ES, EST-SSR; A, ALP; s, SNP.

**Figure 3:**
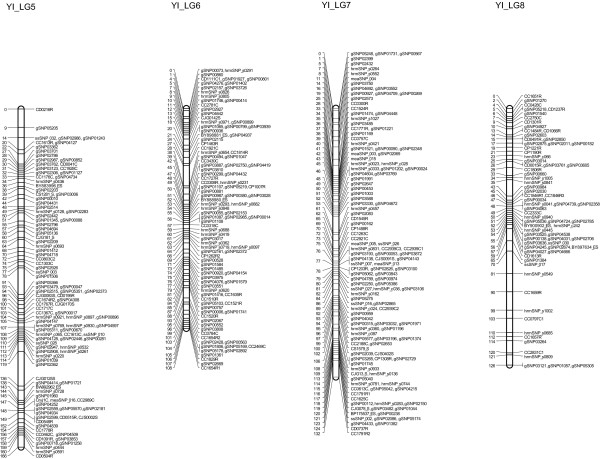
**A linkage map for *C. japonica* derived from the YI pedigree (LG5, LG6, LG7 and LG8)**. Marker names are indicated to the right of the linkage groups. Centimorgan distances (Kosambi) are indicated to the left of each linkage group. Definitions for the labels ssSNP, maeSNP and hrmSNP are provided in the Materials and Methods section. The nature of the other markers is indicated by the last letter of the locus names: C, CAPS; R, RFLP; S, SSR; ES, EST-SSR; A, ALP; s, SNP.

**Figure 4:**
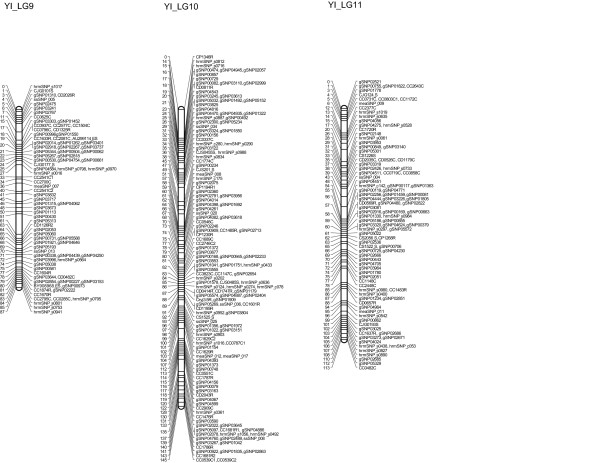
**A linkage map for *C. japonica* derived from the YI pedigree (LG9, LG10 and LG11)**. Marker names are indicated to the right of the linkage groups. Centimorgan distances (Kosambi) are indicated to the left of each linkage group. Definitions for the labels ssSNP, maeSNP and hrmSNP are provided in the Materials and Methods section. The nature of the other markers is indicated by the last letter of the locus names: C, CAPS; R, RFLP; S, SSR; ES, EST-SSR; A, ALP; s, SNP.

Of the 1290 markers that exhibited no segregation distortion, 1279 could be assigned to specific linkage groups; in total, 1262 markers were mapped, including 761 gSNPs, 159 hrmSNPs, 121 CAPSs, 117 RFLPs, 34 SSRs, 33 ssSNPs, 15 meaSNPs, 16 EST-SSRs, 5 SNPs and 1 ALP (Figures 2, 3 and 4). The observed and estimated map lengths were 1405.2 cM (*G_o_*) and 1430.6 cM (*G_e_*), respectively; the observed and estimated map coverages were estimated to be 98.2% (*C_o_*) and 100.0% (*C_e_*), respectively. The maximum observed map distance between two adjacent assigned markers in cM (*X*) was 17.3 cM. Significant deviations from the Poisson distribution of markers were observed for marker intervals of 1 cM, 2 cM, 3 cM, 4 cM, 5 cM, 10 cM and 15 cM (*P *< 0.001), indicating that the 1262 markers used to construct the linkage map for the YI pedigree were not randomly distributed (Additional file 6). Linkage map for the YI pedigree is shown on the TreeGenes database (http://dendrome.ucdavis.edu/cmap/, accession: TG122).

### The construction of a partial linkage map around the *ms1 *locus

19 SSRs were screened to identify markers linked to the *ms1 *gene. Independence testing was conducted using the chi-squared method; two suitable SSRs were found, both of which are on LG9: CJG0101S and CJG0177_S (Additional file 4).

**Figure 5:**
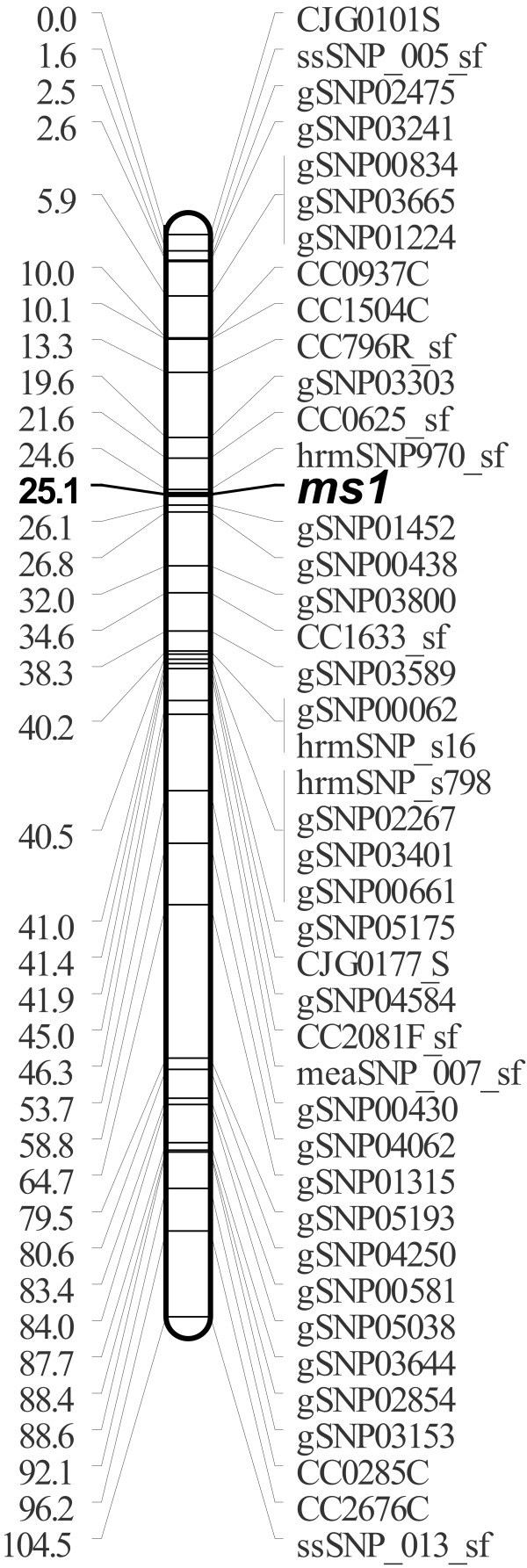
**A partial linkage map around the *ms1 *locus for *C. japonica*, derived from the TO-S pedigree**. Marker names are indicated to the right of the linkage groups. Centimorgan distances (Kosambi) are indicated to the left of each linkage group. The *ms1 *locus is indicated in bold. The SSR and CAPS markers are indicated by the last latter of the locus names: C, CAPS; S, SSR.

A partial linkage map around the *ms1 *locus was constructed for the TO-S pedigree using the markers assigned to LG9 in the YI pedigree. While gSNP markers were not used in the construction of the YI linkage map, those that appeared to be linked to the *ms1 *gene on the basis of the segregation patterns observed in 17 male-infertile TO-S progeny were also used in the construction of the partial map. In total, 42 markers were ultimately mapped in the vicinity of the *ms1 *locus (Figure 5). Three markers (ssSNP_005, meaSNP_007 and ssSNP_013) identified by the candidate gene approach were mapped to LG9 but were not closely linked to the *ms1 *gene (i.e. were separated from it by > 20 cM). The YI map indicated that most of the ssSNP and meaSNP markers considered were associated with other linkage groups. The partial linkage map around the *ms1 *locus was constructed for the TO-S pedigree is shown on the TreeGenes database (http://dendrome.ucdavis.edu/cmap/, accession: TG123).

The marker most tightly linked to the *ms1 *gene was hrmSNP970_sf, which was separated from it by only 0.5 cM in the TO-S pedigree.

## Discussion

### A high-density linkage map for the YI pedigree

The success rate for SNP genotyping (i.e. the SNP conversion rate) with the GoldenGate assay was 81.6% in *Picea glauca *[17], 82.0% in *P. mariana *[17] and 66.9% in *Pinus taeda *[56]; these values are similar to those we observed for *C. japonica *(84.9%), although the evaluation criteria used in this work differed slightly from those used in previous studies. These values are slightly lower than that obtained in crops, e.g. 91.3% in barley [57], 89.0% in soybean [58] and 92.0% in maize [59]. The lower conversion rate for conifers compared to food crops may be due to the complexity of conifer genomes, which would be expected to hamper the development of specific probes for the assay, as suggested by Pavy et al. (2008) [17]. Nonetheless, the high success rate achieved in this work suggests that Illumina's high-throughput GoldenGate SNP genotyping assay is an efficient method for mapping EST-based markers and enriching linkage maps for almost any species.

The GoldenGate assay was used to construct a high-density linkage map of *C. japonica *featuring 1262 markers. The observed map length (*G_o_*) was 1405.2 cM, and the average marker interval was only 1.1 cM. A total of 11 distinct linkage groups were identified, which corresponds to the haploid number of chromosomes in *C. japonica*. The number of mapped markers in *C. japonica *was higher and the average interval between markers was smaller than those reported for other conifers (Table 2). While anonymous markers such as AFLPs were used extensively in previous conifer linkage maps, most of the mapped markers in *C. japonica *were highly informative co-dominant markers derived from EST. The incorporation of EST-based markers into the linkage map should facilitate comparative mapping between *C. japonica *and related species such as *Chamaecyparis obtusa*, which is the second most important forestry tree species in Japan; it has also been shown that around 30% of *C. japonica *EST-based STS markers are directly applicable to *C. obtusa *[60]. In addition, when EST-based markers are identified in a target trait locus, they are generally more effective than other kinds of markers for identifying genes that affect the relevant traits [61,62].

**Table 2:** Comparison of the linkage map constructed in this work to those constructed for other conifers

Species	No. of mapped markers	Markers	No. of linkage groups	Observed map length in cM (Kosambi)	Average distance between markers (cM)	Reference
*Pinus pinaster*	1182	1161 AFLPs, 14 SSRs, 7 ESTs	12	1994	1.7	Ritter et al. 2002

*Picea abies*	755	661 AFLPs, 74 SSRs, 18 ESTPs, the 5S rDNA, the early cone formation	12	2035	2.6	Acheré et al. 2004

*Picea glauca*	821	461 AFLPs, 317 SNPs, 12 SSRs, 31 ESTPs	12	2304	2.8	Pavy et al. 2008

*Picea mariana*	1111	809 AFLPs, 255 SAMPL, 42 SSRs, 5 ESTPs	12	1914	1.7	Kang et al. 2010

*Cryptomeria japonica*	1262	968 SNPs, 121 CAPS, 117 RFLPs, 34 SSRs, 16 EST-SSRs, 1 ALP	11	1405	1.1	This study

The genome length in *C. japonica *estimated in our study is 1430.6 cM (Kosambi). The observed and expected genome coverages were 98.2 (*C_o_*) and 100.0% (*C_e_*), respectively. It thus appears that the *C. japonic*a linkage map developed in this work is almost saturated.

As was shown to be the case in *P. mariana *[55], chi-squared testing indicated that the distribution of markers in the *C. japonica *genome was non-random. This suggests that there are marker-rich and marker-poor regions in the *C. japonica *linkage map. If markers were distributed equally over the genome, they would be expected to concentrate in regions of suppressed recombination such as centromeric regions, in which the map distance between markers becomes shorter than their physical separation, as reported for barley [63] and maize [64]. Feuillet and Keller (2002) [65] suggested that genes are not distributed randomly and there are gene-rich and gene-poor regions in species with large genomes. The non-random distribution of markers in the *C. japonica *linkage map created in this work might thus reflect the distribution of genes in this species, since most of the mapped markers were based on ESTs. To fill in the gaps, it would be desirable to add data on markers for non-coding regions such as genomic microsatellite markers or random genetic markers such as AFLPs.

It seems unlikely that any of the ssSNP and meaSNP markers identified in this work correspond to the *ms1 *gene. It is possible that this is because the genes associated with male gametophyte development and male sterility in other species are not closely related to the *ms1 *gene in *C. japonica*. Alternatively, the putative homologs detected in this study might not be orthologous to those involved in male gametophyte development in other species; instead, they may be paralogous, with similar domains. While one would expect that genes involved in male gametogenesis would exhibit differential expression in male-sterile and male-fertile individuals, and that this difference would be detectable by analysing the microarray expression data, it is possible that the difference may be statistically insignificant owing to the limitations of the methodology. Future studies in this area should aim to address these issues. In addition to the problems related to the identification of a specific sequence corresponding to *ms1*, it should be noted that 217 (80.4%) of the 270 candidate genes could not be located on the linkage map due to a lack of polymorphism in the parents of the YI pedigree or because the sequence data was very complex. The efficiency of the mapping could potentially be improved to address these issues by designing primers that span exon/intron junctions. Once suitable candidate genes have been identified, they should be exploited in studies on the TO-S pedigree.

### A partial linkage map around the *ms1 *locus

Although we were unable to assign a specific sequence for *ms1*, we were able to determine that it is located on LG9 and to construct a partial linkage map around the *ms1 *locus (Figure 5). To our knowledge, this is the first case in which a recessive major gene has been localized on a linkage map in conifers, although they have previously been identified using morphological and biochemical mutants [66-69]. The closest marker to the *ms1 *locus was hrmSNP970_sf; the map distance between the locus and the marker was only 0.5 cM. By using the two closest markers to the *ms1 *locus (hrmSNP970_sf and gSNP01452), the male-sterility or -fertility of 96.6% of the 205 individuals in the TO-S pedigree could be accurately determined. In theory, this will facilitate the selection of individuals that are heterozygous for the *ms1 *gene without needing to create control crosses. The identification of these two adjacent markers will increase the viability of using MAS in the TO-S pedigree. This will make it possible to perform early selection of germinated seedlings, which could be useful in that it would reduce the expenditure of time, labour, and space on the growing of seedlings.

While the two markers will be very useful for TO-S pedigree, it will be necessary to identify the target gene itself to do MAS in other pedigrees. To this end, candidate gene approaches may become more useful as the amount of information on the species increases and techniques improve. An alternative approach for identifying target genes is genome walking using a BAC library. The likelihood of isolating a gene using genome walking depends on the physical distance (per cM) in the target species. On the basis of the estimated genome length calculated from the recombination rates observed in this study (*G_e_*; 1430.6 cM), the average physical distance per cM would be roughly 7.5 Mb. This physical distance per cM in *C. japonica *suggests that the closest markers we identified may be around 3.8 Mb from *ms1 *locus. As such, genome walking would be impractical with current methods, but it would be feasible to isolate the *ms1 *gene by BAC walking if markers lying within 0.1 cM of the target could be identified. We have constructed a BAC library for *C. japonica *that covers 4 times as much of the genome as the marker libraries employed in this work and relates to a mapping population with a large number of individuals (unpublished data). The construction of a more dense linkage map around the *ms1 *gene will greatly facilitate its isolation or that of more tightly-linked markers.

## Conclusions

We have constructed a high-density linkage map for *C. japonica *using expressed sequence-derived co-dominant SNP markers that were primarily genotyped using the GoldenGate assay. A total of 1261 markers were assigned to 11 linkage groups with an observed map length of 1405.2 cM and a mean distance between adjacent markers of 1.1 cM. The number of linkage groups identified matches the basic chromosome number of *C. japonica*. While other conifer linkage maps have largely relied on anonymous markers such as AFLPs, most of the markers mapped for *C. japonica *were highly informative co-dominant markers derived from ESTs. The expected map coverage rate of this constructed linkage map was very high (100.0%), indicating that the linkage map developed in this work is almost saturated. The distribution of the mapped loci on the linkage map for the YI pedigree was not random, as demonstrated by a chi-squared test (*χ*^2 ^= 3233.7, *df *= 13, *P *< 0.001).

Genetic male-sterility in *C. japonica *is known to be controlled by a major recessive gene (*ms1*). We mapped the *ms1 *gene to the 9^th ^linkage group and constructed a partial linkage map around the *ms1 *locus using information from the dense map constructed in this work. A total of 42 markers were located on this partial linkage map. A marker, hrmSNP970_sf that is tightly linked to the *ms1 *gene was identified; the two are separated by only 0.5 cM. The markers linked to *ms1 *identified in this work will facilitate the early selection of male-sterile trees, which should prove useful in *C. japonica *breeding programs.

## Authors' contributions

YM: preparation of manuscript, construction of linkage map, experimental work and genotyping for EST-SSR, HRM and SNP markers; TUI: SNP discovery, assistance with HRM analysis; KU: SNP discovery; NF: Screening of the candidate gene; MS: development of the TO-S mapping population; SU: design of primers for KASPar assays; AM: assistance with sequencing; NT: provision of segregation data for RFLP, CAPS, SSR and ALP markers; HT: discovery of male-sterile tree in *C. japonica*; KS: providing the funding for the screening and genotyping of the candidate gene; YT: supervising the project, providing the funding for genotyping using the SNP array. All authors read and approved the final manuscript.

## Supplementary Material

Additional file 1 **Candidate markers developed from genes with significant similarity to genes related to male gametophyte development and male sterility in other plant species**. Click here for file

Additional file 2 **Candidate markers developed from genes differently expressed between male-fertile and male-sterile individuals**. Click here for file

Additional file 3 **Primer information of hrmSNP markers**. The method used for marker development has previously been reported by Ujino-Ihara et al. (2010). Click here for file

Additional file 4 **Linkage association between microsatellite markers and a male-sterile gene (*ms1*) in *C. japonica***. Click here for file

Additional file 5 **Primer information for BioMark 48.48 Dynamic Array (Fluidigm) using KASPar assays**. Click here for file

Additional file 6 **Results of marker distribution analysis in each marker interval**. Click here for file

## References

[B1] Zhong S Toubia-Rahme H Steffenson BJ Smith KP Molecular mapping and marker-assisted selection of genes for septoria speckled leaf blotch resistance in barley Phytopathology 2006 96 993 999 10.1094/PHYTO-96-0993 18944055

[B2] Jena KK Jeung JU Lee JH Choi HC Brar DS High-resolution mapping of a new brown planthopper (BPH) resistance gene, *Bph18*(*t*), and marker-assisted selection for BPH resistance in rice (*Oryza sativa *L.) Theor Appl Genet 2006 112 288 297 10.1007/s00122-005-0127-8 16240104

[B3] El Mohtar CA Atamian HS Dagher RB Abou-Jawdah Y Salus MS Maxwell DP Marker-assisted selection of tomato genotypes with the *I*-2 gene for resistance to *Fusarium oxysporum *f. sp. *lycopersici *race 2 Plant Disease 2007 91 758 762 10.1094/PDIS-91-6-0758 30780487

[B4] Devey ME Delfino-Mix A Kinloch BB Jr Neale DB Random amplified polymorphic DNA markers tightly linked to a gene for resistance to white pine blister rust in sugar pine Proc Natl Acad Sci USA 1995 92 2066 2070 10.1073/pnas.92.6.2066 11607517 PMC42424

[B5] Harkins DM Johnson GN Skaggs PA Mix AD Dupper GE Devey ME Kinloch BB Jr Neal DB Saturastion mapping of a major gene for resistance to white pine blister rust in sugar pine Theor Appl Genet 1998 97 1355 1360 10.1007/s001220051029

[B6] Liu JJ Ekramoddoullah AKM Hunt RS Zamani A Identification and characterization of random amplified polymorphic DNA markers linked to a major gene (*Cr2*) for resistance to *Cronartium ribicola *in *Pinus monticola* Phytopathology 2006 96 396 399 10.1094/PHYTO-96-0395 18943421

[B7] Wilcox PL Amerson HV Kuhlman EG Liu BH O'Malley DM Sederoff RR Detection of a major gene for resistance to fusiform rust disease in loblolly pine by genomic mapping Proc Natl Acad Sci USA 1996 93 3859 3864 10.1073/pnas.93.9.3859 8632980 PMC39449

[B8] Kondo T Terada K Hayashi E Kuramoto N Okamura M Kawasaki H RAPD markers linked to a gene for resistance to pine needle gall midge in Japanese black pine (*Pinus thunbergii*) Theor Appl Genet 2000 100 391 395 10.1007/s001220050051

[B9] Hayashi E Kondo T Terada K Kuramoto N Kawasaki S Identification of AFLP markers linked to a resistance gene against pine needle gall midge in Japanese black pine Theor Appl Genet 2000 108 1177 1181 15067405 10.1007/s00122-003-1537-0

[B10] Hizume M Kondo T Shibata F Ishizuka R Flow Cytometric Determination of Genome Size in the Taxodiaceae, Cupressaceae sensu stricto and Sciadopityaceae Cytologia 2001 66 307 311 10.1508/cytologia.66.307

[B11] Doležel J Bartosš J Voglmayr H Greilhuber J Nuclear DNA content and genome size of trout and human Cytometry 2003 51 127 128 12541287 10.1002/cyto.a.10013

[B12] Ishizaki T Koizumi K Ikemori R Ishiyama Y Kushibiki E Studies of prevalence of Japanese cedar pollinosis among residents in a densely cultivated area Ann Allergy 1987 58 265 270 3565861

[B13] Taira H Teranishi H Kenda Y A case study of male sterility in sugi (*Cryptomeria japonica*) J Jpn For Soc 1993 75 Japanese with English summary 377 379

[B14] Taira H Saito M Furuta Y Inheritance of the trait of male sterility in *Cryptomeria japonica* J For Res 1999 4 271 273 10.1007/BF02762782

[B15] Saito M Breeding strategy for the pollinosis preventive cultivars of *Cryptomeria japonica *D Don J Jpn For Soc 2010 92 Japanese with English summary 316 323 10.4005/jjfs.92.316

[B16] Tani N Takahashi T Iwata H Yuzuru M Ujino-Ihara T Matsumoto A Yoshimura K Yoshimaru H Murai M Nagasaka K Yoshihiko T A consensus linkage map for sugi (*Cryptomeria japonica*) from two pedigrees, based on microsatellites and expressed sequence tags Genetics 2003 165 1551 1568 14668402 10.1093/genetics/165.3.1551 PMC1462850

[B17] Pavy N Pelgas B Beauseigle S Blais S Gagnon F Gosselin I Lamothe M Isabel N Bousquet J Enhancing genetic mapping of complex genomes through the design of highly-multiplexed SNP arrays: application to the large and unsequenced genomes of white spruce and black spruce BMC Genomics 2008 9 21 10.1186/1471-2164-9-21 18205909 PMC2246113

[B18] Ujino-Ihara T Yoshimura K Ugawa Y Yoshimaru H Nagasaka K Tsumura Y Expression analysis of ESTs derived from the inner bark of *Cryptomeria japonica* Plant Mol Biol 2000 43 451 457 10.1023/A:1006492103063 11052197

[B19] Ujino-Ihara T Taguchi Y Yoshimura K Tsumura Y Analysis of expressed sequence tags derived from developing seed and pollen cones of *Cryptomeria japonica* Plant Biol 2003 5 600 607 10.1055/s-2003-44690

[B20] Ujino-Ihara T Kanamori H Yamane H Taguchi Y Namiki N Mukai Y Yoshimura K Tsumura Y Comparative analysis of expressed sequence tags of conifers and angiosperms reveals sequences specifically conserved in conifers Plant Mol Biol 2005 59 895 907 10.1007/s11103-005-2080-y 16307365

[B21] Futamura N Ujino-Ihara T Nishiguchi M Kanamori H Yoshimura K Sakaguchi M Shinohara K Analysis of expressed sequence tags from *Cryptomeria japonica *pollen reveals novel pollen-specific transcripts Tree Physiol 2006 26 1517 1528 10.1093/treephys/26.12.1517 17169891

[B22] Futamura N Totoki Y Toyoda A Igasaki T Nanjo T Seki M Sakaki Y Mari A Shinozaki K Shinohara K Characterization of expressed sequence tags from a full-length enriched cDNA library of *Cryptomeria japonica *male strobili BMC Genomics 2008 9 383 10.1186/1471-2164-9-383 18691438 PMC2568000

[B23] Yoshida K Nishiguchi M Futamura N Nanjo T Expressed sequence tags from *Cryptomeria japonica *sapwood during the drying process Tree Physiol 2007 27 1 9 10.1093/treephys/27.1.1 17169901

[B24] Tsumura Y Suyama Y Yoshimura K Shirato N Mukai Y Sequence-tagged-sites (STSs) of cDNA clones in *Cryptomeria japonica *and their evaluation as molecular markers in conifers Theor Appl Genet 1997 94 764 772 10.1007/s001220050476

[B25] Nikaido AM Ujino T Iwata H Yoshimura K Yoshimura H Suyama Y Murai M Nagasaka K Tsumura Y AFLP and CAPS linkage maps of *Cryptomeria japonica* Theor Appl Genet 2000 100 825 831 10.1007/s001220051358

[B26] Iwata H Ujino-Ihara T Yoshimura K Nagasaka K Mukai Y Tsumura Y Cleaved amplified polymorphic sequence markers in sugi, *Cryptomeria japonica *D. Don, and their locations on a linkage map Theor Appl Genet 2001 103 881 895 10.1007/s001220100732

[B27] Uchiyama K Ujino-Ihara T Ueno S Taguchi Y Futamura N Shinohara K Tsumura Y Single nucleotide polymorphisms in Cryptomeria japonica: their discovery and validation for genome mapping and diversity studies in press

[B28] Yoshii E Study for the characteristics and applications of nuclear male sterility in *Cryptomeria japonica *D. Don PhD thesis 2007 Niigata University, Graduate School of Science and Technology (in Japanese)

[B29] Tsumura Y Yoshimura K Tomaru N Ohba K Molecular phylogeny of conifers using RFLP analysis of PCR-amplified specific chloroplast genes Theor Appl Genet 1995 91 1222 1236 10.1007/BF00220933 24170050

[B30] Ujino-Ihara T Taguchi Y Moriguchi Y Tsumura Y An efficient method for developing SNP markers based on EST data combined with high resolution melting (HRM) analysis BMC Res Notes 2010 3 51 10.1186/1756-0500-3-51 20193087 PMC2850910

[B31] Moriguchi Y Ueno S Ujino-Ihara T Futamura N Matsumoto A Shinohara K Tsumura Y Characterization of EST-SSRs from *Cryptomeria japonica* Conserv Genet Resour 2009 1 373 376 10.1007/s12686-009-9086-8

[B32] Xu H Knox R Taylor P Singh M *Bcp1*, a gene required for male fertility in *Arabidopsis* Proc Natl Acad Sci USA 1995 92 2106 2110 10.1073/pnas.92.6.2106 7892232 PMC42432

[B33] Aarts MGM Hodge R Kalantidis K Florack D Wilson ZA Mulligan BJ Stiekema WJ Scott R Pereira A The *Arabidopsis MALE STERILITY *2 protein shares similarity with reductases in elongation/condensation complexes Plant J 1997 12 615 623 10.1046/j.1365-313X.1997.d01-8.x 9351246

[B34] Rubinelli P Hu Y Ma H Identification, sequence analysis and expression studies of novel anther-specific genes of *Arabidopsis thaliana* Plant Mol Biol 1998 37 607 619 10.1023/A:1005964431302 9687065

[B35] Paxson-Sowders DM Dodrill CH Owen HA Makaroff CA *DEX1*, a novel plant protein, is required for exine pattern formation during pollen development in *Arabidopsis* Plant Physiol 2001 127 1739 1749 10.1104/pp.010517 11743117 PMC133577

[B36] Wilson ZA Morroll SM Dawson J Swarup R Tighe PJ The *Arabidopsis MALE STERILITY 1 (MS) *gene is a transcriptional regulator of male gametogenesis, with homology to the PHD-finger family of transcription factors Plant J 2001 28 27 39 10.1046/j.1365-313X.2001.01125.x 11696184

[B37] Gupta R Ting JT Sokolov LN Johnson SA Luan S A tumor suppressor homolog, *AtPTEN1*, is essential for pollen development in *Arabidopsis* Plant Cell 2002 14 2495 2507 10.1105/tpc.005702 12368500 PMC151231

[B38] Higginson T Li SF Parish RW *AtMYB103 *regulates tapetum and trichome development in *Arabidopsis thaliana* Plant J 2003 35 177 192 10.1046/j.1365-313X.2003.01791.x 12848824

[B39] Honys D Twell D Transcriptome analysis of haploid male gametophyte development in *Arabidopsis* Genome Biol 2004 5 R85 10.1186/gb-2004-5-11-r85 15535861 PMC545776

[B40] Preston J Wheeler J Heazlewood J Li SF Parish RW *AtMYB32 *is required for normal pollen development in *Arabidopsis thaliana* Plant J 2004 40 979 995 10.1111/j.1365-313X.2004.02280.x 15584962

[B41] Robertson WR Clark K Young JC Sussman MR An *Arabidopsis thaliana *plasma membrane proton pump is essential for pollen development Genetics 2004 168 1677 1687 10.1534/genetics.104.032326 15579716 PMC1448765

[B42] Sorensen A-M Krober S Unte US Huijser P Dekker K Saedler H The *Arabidopsis ABORTED MICROSPORES (AM) *gene encodes a MYC class transcription factor Plant J 2003 33 413 423 10.1046/j.1365-313X.2003.01644.x 12535353

[B43] Cao J Yu X Ye W Lu G Xiang X Functional analysis of a novel male fertility *CYP86MF *gene in Chinese cabbage (*Brassica campestris *L. ssp. *chinensis* makino) Plant Cell Rep 2006 24 715 723 10.1007/s00299-005-0020-6 16075226

[B44] Tsuchiya T Toriyama K Ejiri S Hinata K Molecular characterization of rice genes specifically expressed in the anther tapetum Plant Mol Biol 1994 26 1737 1746 10.1007/BF00019488 7858214

[B45] Lee S Jung KH An G Chung YY Isolation and characterization of a rice cysteine protease gene, *OsCP1*, using T-DNA gene-trap system Plant Mol Biol 2004 54 755 765 15356393 10.1023/B:PLAN.0000040904.15329.29

[B46] Moritoh S Miki D Akiyama M Kawahara M Izawa T Maki H Shimamoto K RNAi-mediated silencing of *OsGEN-L (OsGEN-like)*, a new member of the RAD2/XPG nuclease family, causes male sterility by defect of microspore development in rice Plant Cell Physiol 2005 46 699 715 10.1093/pcp/pci090 15792960

[B47] Atanassov I Russinova E Antonov L Atanassov A Expression of an anther-specific chalcone sythase-like gene is correlated with uninucleate microspore development in *Nicotiana sylvestris* Plant Mol Biol 1998 38 1169 1178 10.1023/A:1006074508779 9869422

[B48] Kapoor S Kobayashi A Takathuji H Silencing of the tapetum-specific zinc finger gene *TAZ1* causes premature degneration of tapetum and pollen abortion in Petunia Plant Cell 2002 14 2353 2367 10.1105/tpc.003061 12368491 PMC151222

[B49] Altschul SF Madden TL Schaffer AA Zhang J Zhang Z Miller W Lipman DJ Gapped BLAST and PSI-BLAST: a new generation of protein database search programs Nucleic Acids Res 1997 25 3389 3402 10.1093/nar/25.17.3389 9254694 PMC146917

[B50] Van Ooijen JW Voorrips RE JoinMap version 3.0, software for the calculation of genetic linkage maps Plant Research International 2001 Wageningen, The Netherlands

[B51] Kosambi DD The estimation of map distances from recombination values Ann Eugen 1944 12 172 175

[B52] Voorrips RE MapChart: software for the graphical presentation of linkage maps and QTLs J Hered 2002 93 77 78 10.1093/jhered/93.1.77 12011185

[B53] Chakravarti A Lasher LK Reefer JE A maximum-likelihood method for estimating genome length using genetic linkage data Genetics 1991 128 175 182 2060775 10.1093/genetics/128.1.175 PMC1204446

[B54] Bishop DT Cannings C Skolnick M Williamson JA Weir BS The number of polymorphic DNA clones required to map the human genome Statical analysis of DNA sequence data 1983 New York: Marcel-Dekker 181 200

[B55] Kang BY Mann IK Major JE Rajora OP Near-saturated and complete genetic linkage map of black spruce (*Picea mariana*) BMC Genomics 2010 11 515 10.1186/1471-2164-11-515 20868486 PMC2997009

[B56] Eckert AJ Pande B Ersoz ES Wright MH Rashbrook VK Nicolet CM Neal DB High-throughput genotyping and mapping of single nucleotide polymorphisms in loblolly pine (*Pinus taeda *L.) Tree Genet Genomes 2009 5 225 234 10.1007/s11295-008-0183-8

[B57] Rostoks N Ramsay L MacKenzie K Cardle L Bhat PR Roose ML Svensson JT Stein N Varshney RK Marshall DF Graner A Close TJ Waugh R Recent history of artificial outcrossing facilitates whole-genome association mapping in elite inbred crop varieties Proc Natl Acad Sci USA 2006 103 18656 18661 10.1073/pnas.0606133103 17085595 PMC1693718

[B58] Hyten D Song Q Choi IY Yoon MS Specht JE Matukumalli LK Nelson RL Shoemaker RC Young ND Cregan PB High-throughput genotyping with the GoldenGate assay in the complex genome of soybean Theor Appl Genet 2008 116 945 952 10.1007/s00122-008-0726-2 18278477

[B59] Yan J Yang X Shah T Sánchez-Villeda H Li J Warburton M Zhou Y Crouch JH Xu Y High-throughput SNP genotyping with the GoldenGate assay in maize Mol Breed 2010 25 441 451 10.1007/s11032-009-9343-2

[B60] Matsumoto A Tsumura Y Evaluation of cleaved amplified polymorphic sequence markers for *Chamaecyparis obtusa *based on expressed sequence tag information from *Cryptomeria japonica* Theor Appl Genet 2004 110 80 91 10.1007/s00122-004-1754-1 15549233

[B61] Wang Z Taramino G Yang D Liu G Tingey SV Miao GH Wang GL Rice ESTs with disease-resistance gene- or defense-response gene-like sequences mapped to regions containing major resistance genes or QTLs Mol Gen Genomics 2001 265 302 310 10.1007/s004380000415 11361341

[B62] Szűcs P Karsai I von Zitzewitz J Mészáros K Cooper LLD Gu YQ Chen THH Hayes PM Skinner JS Positional relationships between photoperiod response QTL and photoreceptor and vernalization genes in barley Theor Appl Genet 2006 112 1277 1285 10.1007/s00122-006-0229-y 16489429

[B63] Qi X Stam P Lindhout P Use of locus-specific AFLP markers to construct a high-density molecular map in barley Theor Appl Genet 1998 96 376 384 10.1007/s001220050752 24710875

[B64] Castiglioni P Ajmone-Marsan P van Wijk R Motto M AFLP markers in a molecular linkage map of maize: codominant scoring and linkage group distribution Theor Appl Genet 1999 99 425 431 10.1007/s001220051253 22665174

[B65] Feuillet C Keller B Comparative genomics in the grass family: molecular characterization of grass genome structure and evolution Ann Bot 2002 89 3 10 10.1093/aob/mcf008 12096816 PMC4233775

[B66] Chiba S Inheritance of colouring of needles in the fall of sugi (*Cryptomeria japonica *D. Don) J Jpn For Soc 1953 35 Japanese 286 289

[B67] Ohba K Murai M Sugimura G Saito M Okamoto K Watanabe M Noguchi T Studies on variation of forest trees (III): Cross-fertility between Kuma-sugi and some other cutting varieties of sugi (*Cryptomeria japonica*), growth of the F1 seedlings, and two single recessive genes detected in Kuma-sugi J Jpn For Soc 1967 49 Japanese 361 367

[B68] Ohba K Murai M Recessive genes producing albino- and light green seedlings in sugi, *Cryptomeria japonica *D Don J Jpn For Soc 1971 53 Japanese 177 180

[B69] Yasue M Ogiyama K Suto S Tsukahara H Miyahara F Ohba K Geographical differentiation of natural *Cryptomeria *stands analyzed by diterpene hydrocarbon constituents of individual trees J Jpn For Soc 1987 69 Japanese 152 156

